# Internet Use among Older Adults in the Era of COVID-19 in China: Challenges and Opportunities

**DOI:** 10.14336/AD.2022.1006

**Published:** 2023-06-01

**Authors:** Qingqing Yin, Xinyu Wang, Renjun Lv, Shangbin Li, Xunyao Hou, Jianchun Wang

**Affiliations:** ^1^Department of Geriatric Neurology, Shandong Provincial Hospital Affiliated to Shandong First Medical University, Jinan, Shandong, China.; ^2^Department of Geriatrics, Shandong Provincial Hospital Affiliated to Shandong First Medical University, Jinan, Shandong, China.; ^3^Department of Geriatric Cardiology, Shandong Provincial Hospital Affiliated to Shandong First Medical University, Jinan, Shandong, China.

**To the editor,**

The coronavirus disease (COVID-19) pandemic caused by severe acute respiratory syndrome coronavirus 2 (SARS-CoV-2) globally has affected the health system as well as economic and social life unpredictably [1]. The Chinese Center for Disease Control and Prevention summarized the characteristics of 72,314 cases of the COVID-19 in China [2], noting that 3% of patients were 80 years of age or older, with a mortality rate of 8% in patients aged 70-79 years and 14.8% in cases aged 80 years or older. The COVID-19 pandemic has taken a great risk to the lives and health of the elderly.

According to “The 50^th^ Statistical Report on the Development of China’s Internet”, Internet users in China reached 1,051 million by June 2022, and 11.3% of them are older than 60. The number of Internet users in elderly keeps increasing, accounting for 20% of the elderly population. However, there is still a big gap in digital inclusion with other age groups. The “digital divide” was highlighted, it brought challenges and changes in the lives of the elderly. The Chinese Academy of Social Sciences Institute for Social Development Strategy recently released the report “Research on the Aging of the Internet in the Post-epidemic Era”, which showed the changes in the digital life of the elderly in the post-epidemic era. The most significant change is that the elderly use social tools more frequently. 95.09% of the elderly believe that it is very necessary to learn network operations after the epidemic. 93.36% of the elderly think they can learn to surf the Internet with smart phones. The report shows that since 2017, the amount of WeChat pay for the elderly group has basically increased in a straight line, taking the first quarter 2017 as the benchmark (100%), and the growth rate was 5,227% in the second quarter 2021. However, in the prevention and control of the epidemic in China, the elderly encounter difficulties in their daily life such as travel, medical treatment, and consumption. Addressing the "digital divide" will enable them to better adapt to and integrate into a smart society.

The elderly may have multiple geriatric diseases, and their online writing and reading skills have declined. So, they are required to take a learning cost for them to be proficient in the functions within the application of the Internet. A series of operations such as registering an account, user login, opening the application, and clicking the sweeping code need to be completed within a few minutes. Moreover, online medical, online shopping, mobile payment, and other functions are still relatively unfamiliar for the elderly, and often become the group of network telecom fraud, which greatly affects the quality of life of the elderly and life satisfaction. Whether the outbreak of the COVID-19 pandemic or the rapid spread of the internet, it is a huge change in the living environment for the elderly, they are less able to cope with uncertainty and new things [3]. In the COVID-19 pandemic, many elderly people do not have smartphones, and even though their children have equipped them with smartphones, they do not know how to operate them. For these elderly people, “scanning code difficulty” has become a practical problem in front of travel and life. In addition, the elderly may have a heightened susceptibility to its adverse consequences, and they may experience a loss of usual social support, such as family members visiting [3]. This abrupt physical threat and loss of social resources may increase the risk for loneliness, isolation, and depression among elderly people [5]. Meanwhile, with the rapid spread of misinformation or fake news through social media platforms and other outlets, elderly people with limited ability to distinguish the truth from false information on the internet. They are easier to be affected by misinformation.

In November 2020, the General Office of the State Council issued “the Implementation Plan on Effectively Solving the Difficulties of the Elderly in Using Smart Technology” to effectively solve the difficulties they encounter in their daily life such as travel, medical treatment, and consumption. First, for groups such as the elderly, alternative measures can be taken such as registering with valid identity documents, passing with paper certificates, and presenting "communication travel cards" as auxiliary travel proof. Localities and places where conditions permit should set up "no health code channels" for the elderly who do not use smartphones. Secondly, in order to effectively solve the problem of the elderly not being able to access online services using intelligent technology, a number of facilities such as convenient consumer service centers and service stations for the elderly in communities have been built and renovated. In addition, medical institutions, related enterprises should improve the telephone, network, on-site and other ways of appointment registration, so their family, relatives and friends, family doctors and other generation of elderly can make appointment registration easy. At last, China government has issued a series of policies to promote "Internet + Healthcare" services for the elderly, to provide the elderly common diseases, chronic diseases, and follow-up management services. The process of online medical services has been simplified, and voice guidance and manual consultation services have been provided for the elderly. The government will promote the use of multi-media such as ID cards, social security cards and electronic medical insurance certificates for medical services and encourage the application of facial recognition and other technologies in medical treatment scenarios. At the same time, smart terminal products such as mobile phones are promoted for aging transformation, so that they have large screen, large font, large volume, large battery capacity, simple operation, and other characteristics convenient for the elderly to use. The government will actively develop intelligent accessories, smart homes, health monitoring, elderly care, and other intelligent terminal products.

The normalization of the COVID-19 pandemic prevention and control has exposed shortcomings in internet use for the elderly, but it also brings with it the opportunities in “Internet + Healthcare”. A truly good and responsible Internet application that is suitable for aging means gradually closing the “digital divide” rather than exacerbating it. It is to fully respect and meet the internal needs of the elderly who are eager to be recognized and accepted, and willing to learn and adapt to the Internet society, instead of ignoring and denying their needs. It is to continuously improve the humanization and age-appropriate level of Internet applications, to help the elderly better adapt to and fully integrate into the social environment where intelligent services are widely used, rather than to isolate some elderly people from the intelligent service environment through the “digital divide”. Finally, the elderly need to improve their initiative to integrate into the Internet society. We need to encourage the elderly to adapt to the new era through government propaganda, television broadcasts, community street activities and other traditional ways, and give them attention and encouragement. Let these new technologies and operations enter the world of the elderly in an appropriate and gentle way, so that they can exert their initiative and actively connect with modern society and the Internet.

## References

[1] Wu Y, Ho W, Huang Y, et al. (2020). SARS-CoV-2 is an appropriate name for the new coronavirus. Lancet, 395:949-950. 10.1016/S0140-6736(20)30557-2 PMC7133598 32151324

[2] Wu ZY, McGoogan JM (2020). Characteristics of and important lessons from the coronavirus disease 2019 (COVID-19) outbreak in China: summary of a report of 72314 cases from the Chinese center for disease control and prevention. JAMA, 323:1239-1242 32091533 10.1001/jama.2020.2648

[3] Lv R, Yin Q (2021). Prevention and Management of COVID-19 in Patients with Dementia Living at Home: Experience from China. Aging Dis, 12(3):684-687. 34094632 10.14336/AD.2021.0403 PMC8139209

[4] Garnier-Crussard A, Forestier E, Gilbert T, Krolak-Salmon P (2020). Novel Coronavirus (COVID-19) Epidemic: What Are the Risks for Older Patients? J Am Geriatr Soc, 68:939-940. 32162679 10.1111/jgs.16407 PMC7228326

[5] Armitage R, Nellums LB (2020). COVID-19 and the consequences of isolating the elderly. Lancet Public Health, 5:e256. 32199471 10.1016/S2468-2667(20)30061-X PMC7104160

[6] Ghebreyesus TA (2020). Addressing mental health needs: An integral part of COVID-19 response. World Psychiatry, 19(2), 129-130. 32394569 10.1002/wps.20768 PMC7214944

[7] Yin Q, Liu X, Huang C, Bi W, Zhou R, Lv R (2022). Effect of COVID-19 on Geriatric Medical Services in China. Aging Dis, 13:4-7. 35111357 10.14336/AD.2021.0703 PMC8782555

